# Evaluation of Masticatory Muscles in Adult Patients with Maxillary Hypoplasia Treated with Surgically Assisted Rapid Maxillary Expansion (SARME): A Retrospective Study

**DOI:** 10.3390/jcm12020607

**Published:** 2023-01-12

**Authors:** Andrea Abate, Valentina Lanteri, Loris Marcolongo, Luca Solimei, Cinzia Maspero

**Affiliations:** 1 Fondazione IRCCS Cà Granda, Ospedale Maggiore Policlinico, 20142 Milan, Italy; 2 Department of Biomedical Surgical and Dental Sciences, University of Milan, 20142 Milan, Italy; 3 Department of Surgical Science and Integrated Diagnostics (DISC), University of Genova, 16126 Genova, Italy

**Keywords:** malocclusion, SARME, EMG, maxillary expansion

## Abstract

Aim: The aim of the present study was to investigate modifications in electromyographic activity of temporal and masseter muscles before and after surgically assisted rapid maxillary expansion (SARME) in adult subjects. Materials and Methods: Data from 20 patients with unilateral posterior crossbite were selected retrospectively from the Orthodontics Department of the University of Genoa and the Department of Biomedical Surgical and Dental Sciences of the University of Milan, Fondazione IRCCS Ca’ Granda, Ospedale Maggiore Policlinico Milan. Inclusion criteria were set as skeletal class I; adult patients (age > 18); good general health; patients with a transverse maxillary deficiency with unilateral posterior crossbite and maxillary constriction ≥ 5 mm; Superficial electromyographic (EMG) examinations at T0 and T1. Exclusion criteria were smoking, metabolic bone diseases (e.g., hyperparathyroidism, vitamin C deficiency), chronic use of corticoids before or during treatment, parafunctional habits (e.g., bruxism), and temporomandibular joint dysfunction. The Shapiro–Wilk test was performed to check whether the data were normally distributed. Differences for each variable before and after SARME were analyzed with a paired *t*-test (*p* < 0.05). Results: The statistical analysis demonstrated no statistically significant differences between the EMG values taken before and after SARME regarding the standardized electrical activity of the masticatory muscles (masseter and anterior temporalis (*p* > 0.05)). Conclusions: Considering the specific conditions of this study, it can be concluded that SARME did not alter the EMG activity of the masseter and temporal muscles. The present study has shown that the masticatory musculature evaluated after approximately 8 months of therapy can adapt well to SARME.

## 1. Introduction

Maxillary hypoplasia is a frequent malocclusion associated with various clinical conditions. These include upper dental crowding, posterior crossbite, and obstruction of the nasal airway, which affects occlusal stability, swallowing, aesthetics, and physiological stomatognathic functions [1]. The incidence of maxillary hypoplasia in the entire population is estimated at 9.4% [2,3]. Moreover, 13–24% of the European infant population is affected by complete posterior crossbite, compared to 7% of Americans and 1/2% of African children [4].

The aim of the treatment for maxillary hypoplasia is to increase the maxillary width and the dental arch perimeter and this can be achieved through the disjunction of the median palatine suture [5,6,7]. This type of therapy is strictly related to the age of the patients [8].

Rapid maxillary expansion (RME) is an orthopedic procedure for the resolution of maxillary constriction in growing patients [9], whereas surgically assisted rapid maxillary expansion (SARME) is a treatment alternative for adult patients with a mature skeletal structure whose ossified sutures are resistant to orthopedics forces [10]. As reported by several authors, the age limit for orthopedic maxillary expansion is 14–18 years [11,12].

In adult patients, an orthodontic palatal expansion treatment can produce several unwanted effects such as an excessive increase of the tipping of the first upper molars accompanied by extrusion, root resorption, alveolar cortical bone bending, pain, and little or no orthopedic effect [13,14]. SARME treatment is the method of choice and its effectiveness has been demonstrated in the literature [15,16]. The facial skeleton is characterized by a complex structure and mechanics, for this reason, different methods of osteotomy and corticotomy of the mid-face have been developed [17,18]. SARME represents a proper treatment for the correction of maxillary transverse deficiencies in adult subjects, whereas, in cases of maxillary deficiencies associated with alterations on sagittal and vertical planes, the multi-segmental Le Fort I osteotomy is preferred thus solving the whole malocclusion in a single surgical proceeding [19].

The assessment and recording of the electric activity generated by skeletal muscles are essential for studying the performance of facial musculature as it is directly linked to the development of malocclusions [20].

Superficial electromyographic (EMG) examinations are used in many branches of medicine, such as dentistry, speech therapy, and physical and postural therapy, with the aim of obtaining a more accurate diagnosis and prognosis and re-evaluating muscle performance during various stages of treatment [21].

Important data on the functional impact caused by morphological discrepancies can be provided by the EMG examination, which allows the evaluation of the effects produced by treatments to correct occlusal problems [22,23].

A small number of studies evaluated muscular response to maxillary expansion treatment, and the findings are not always in agreement. Some authors electromyographically examined the contraction of the masticatory muscles (temporal and masseter muscles) in patients with crossbite, concluding that, when a correct occlusal relation is obtained, a correct muscle pattern can be achieved [24,25]. Some authors reported that RME influenced the electromyographic activities of the superficial masseter and anterior temporalis muscles during chewing and swallowing with a significant increase [26]. Michelotti et al. [27] affirmed that the treatment of unilateral posterior crossbite using RME did not produce a more symmetric activity of the masticatory muscles.

To date, the electromyographic activity of masticatory muscles after a maxillary expansion has been mainly assessed after RME therapy.

Although SARME is the treatment of choice for the resolution of maxillary transverse deficiency with unilateral or bilateral posterior crossbite in adult patients, the literature is lacking studies assessing the behavior of the masticatory muscles after this therapy.

Thus, the aim of this investigation was to evaluate the electromyographic activity of the masseter and temporalis muscles in adult patients affected by unilateral posterior crossbite before and after SARME.

## 2. Materials and Methods

This longitudinal investigation has been conducted using superficial electromyographic (EMG) examinations of patients treated at the Orthodontics Department of the University of Genoa and the Department of Biomedical Surgical and Dental Sciences of the University of Milan, Fondazione IRCCS Ca’ Granda, Ospedale Maggiore Policlinico Milan. The Ethical Committee of the Fondazione IRCCS Ca’ Granda, Ospedale Maggiore, Milan, Italy (protocol n.573/15) approved the present study. The study protocol was performed in accordance with the principles of the Declaration of Helsinki for medical research involving human subjects. Written informed consent was gathered from all patients to permit us to utilize data for research investigations.

### 2.1. Sample Selection and Inclusion Criteria

Data from patients who underwent SARME were collected and the following inclusion criteria were set: skeletal class I patients based on Steiner ANB angle [28]; Caucasian ethnicity; adult patients (age > 18); good general health; patients with a transverse maxillary deficiency with unilateral posterior crossbite and maxillary constriction ≥ 5 mm; absence of complications during or after the surgical intervention; EMG examinations taken no more than 1 month before SARME and a second EMG exam taken when the appliance was removed. Exclusion criteria were metabolic bone diseases, smoking, temporomandibular joint disorders, chronic use of corticoids during or before treatment, and parafunctional habits.

Based on the aforementioned inclusion and exclusion criteria, 20 patients (10 women and 10 men, mean age 27.3 ± 8.4 years) presenting unilateral posterior crossbite who had undergone SARME surgery and neuromuscular activity assessment were included.

### 2.2. Surgical Procedure

SARME was executed with a Hyrax palatal expander cemented the day before surgery.

The surgery was performed for all patients by the same team and with the same surgical procedure as described by Bell and Jacobs [29].

**Figure 1:**
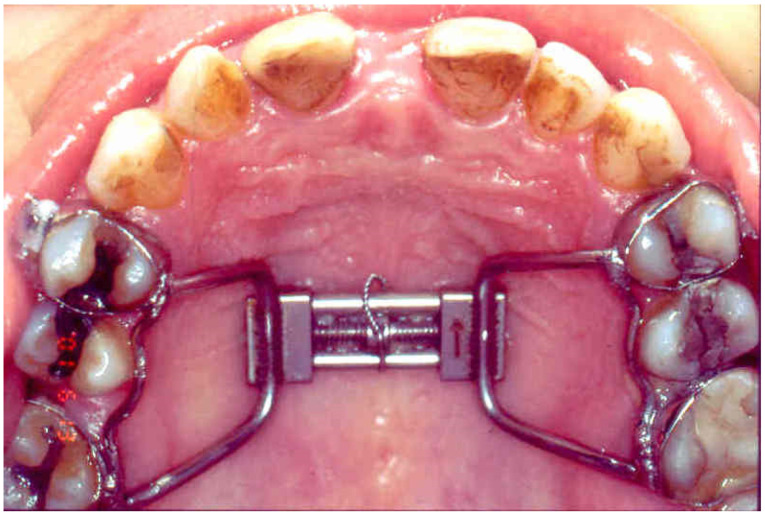
Hyrax expander cemented on the upper first molars and premolars.

The surgical technique requires the same bone cutting used for a Le Fort I osteotomy. A first horizontal incision was made ranging from the first molar to the canine. This incision was done above the mucogingival junction and due to the mucoperiosteal elevation allowed the lateral and anterior walls of the maxilla to be exposed. At this point, a horizontal osteotomy was made from the pyriform rim to the zygomatic buttress. The osteotomy was performed with a micro-reciprocating saw about 5 mm above the tooth apices. A second vertical incision was then performed at the level of the frenulum of the upper lip with the aim of permitting access to the planned site for the interincisal osteotomy which was performed between the roots of the upper central incisors. Once the interincisal osteotomy had been performed, an immediate expansion of the maxilla was obtained with Sverzut’s chisel. At this point in the surgical procedure, the previously cemented orthodontic appliance was activated to achieve the separation of the maxillary bone. The appliance was a Hyrax RME appliance. Bands were placed on the premolars and maxillary first molars. After a 7-day latency period, patients were asked to activate the screw by 0.25 mm twice daily. The patients were monitored once a week until the planned expansion was achieved (Figure 1). Except for hematoma and swelling, no postoperative complications were observed.

### 2.3. EMG Analysis

The EMG activities of the masticatory muscles (anterior temporal and masseter muscles) were detected in all subjects before SARME (T0), and when the appliance was removed (T1) to assess the muscular stability in static conditions [30]. The appliance remained in situ for a period ranging between 6 and 9 months

All analyses were performed in the absence of the palatal expander to avoid interference (at T0 before cementing; at T1 the day after removing it). The head was positioned in the natural head position. All the electromyographic exams were conducted in an undisturbed room.

**Figure 2:**
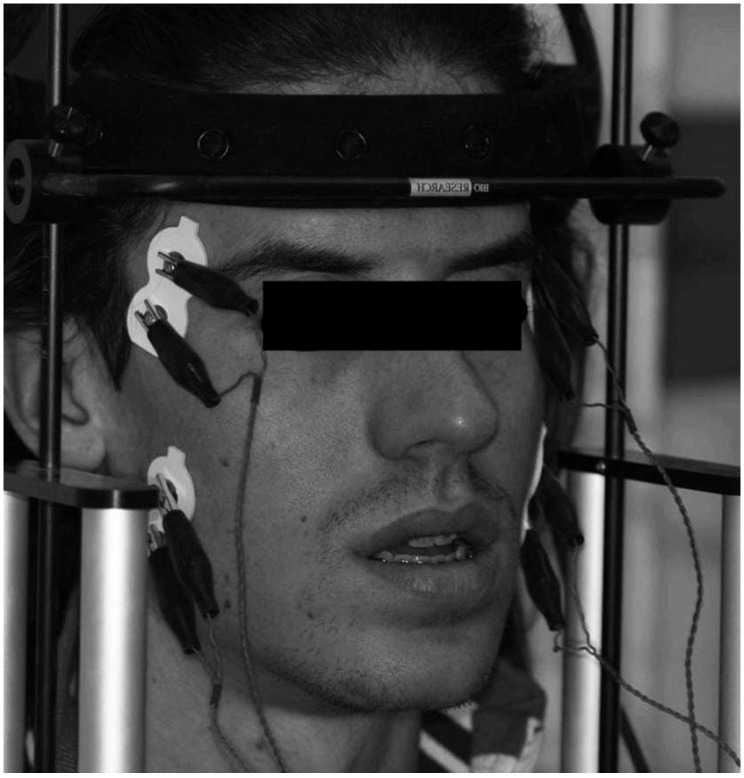
Positioning of the bipolar electrodes in the temporalis muscles and masseter muscles.

The skin of the face was cleaned using a wad soaked in Neoxinal (0.5% chlorhexidine in ≥70% alcohol) and disposable silver/silver chloride bipolar electrodes (Duo-Trode; Myo-Tronics Inc., Seattle, WA, USA) were positioned according to the recommendations of SENIAM (Surface EMG for Non-Invasive Assessment of Muscles) and then a reference electrode was located on the forehead for the electromyographic acquisition [31] (Figure 2).

Electromyographic activity was recorded using a computerized instrument (Freely; De Gotzen srl, Legnano, Milano, Italy), capable of recording, digitizing, amplifying, and filtering the analogical EMG signal [32]. Thanks to a differential amplifier, the electromyographical signals were analogically amplified and digitized (using a gain of 150, theoretical resolution 16 1 V, that is ±14 mV, 12 b resolution, 2230 Hz A/D sampling frequency, peak-to-peak input range 28 mV). Then the analog EMG signal was filtered by analog and digital filtering.

The average electrical activity of the muscles every 25 ms were considered, with muscle activity evaluated as the average square of the root (RMS) of the amplitude (unit: 1 V).

Two electromyographic recordings were made at T0 and T1: the first involved the recording of maximum voluntary clamping (MVC) performed on two 10 mm thick cotton rolls placed between the mandibular posterior teeth and was called “standardization recording”; the second, called “experimental recording”, involved MVC recording performed without the interposition of the cotton rolls. Both recordings had a duration of 5 s, but the software automatically selected only the 3 s with the most stable EMG signal.

All electromyographic values recorded during the “experimental recordings” were expressed as a percentage of the mean potentials recorded during the standardization test carried out on cotton rolls (unit: μV/μV × 100). All consequent calculations were done with the standardized potentials. Thanks to this standardization, there should be no variability due to electrode positioning or skin, and electrode impedance and relative percentage EMG values should be influenced only by the occlusal surfaces [30,33]. The parameters assessed during the EMG analysis are synthesized in Table 1.

### 2.4. Statistical Analysis

The G*Power free software (version 3.1.9.4, Franz Faul, Universitat Kiel, Kiel, Germany) was initially used to obtain data for the power analysis calculation. The mean values of the T0–T1 changes and the relative standard deviation of the RMS normalized means (microvolts) related to the masseter muscle in dental clenching reported by Sverzut et al. [34] were used.

The power analysis evaluation reported that to reach 95% power, 4 patients were necessary to have a statistically significant comparison of the data. As reported above, the authors were able to select 20 subjects.

IBM SPSS Statistics ver. 25.0 software (IBM Co., Armonk, NY, USA) was used to execute the statistical analysis. The Shapiro–Wilk test showed a Gaussian distribution of the data and, thus, parametric tests were used. Data reported in the statistical analysis were reported as mean ± standard deviation (SD) of each EMG index. Differences for each variable before and after SARME were analyzed with a paired T-test and the level of statistical significance was set at *p* < 0.05.

## 3. Results

The statistical analysis did not demonstrate statistically significant differences between the EMG values taken before and after SARME regarding the standardized electrical activity of the masticatory muscles (anterior temporalis and masseter) (*p* > 0.05) (Table 2).

Analyzing the data in Table 2, the EMG indices obtained at T0 show that all patients had good muscle balance as all values were within reference ranges. After treatment, all EMG indices obtained remained within the reference values showing that muscle balance after SARME was maintained. The values obtained from POCm, POCt, TC, and Ac showed that at T1 as well as at T0 there was good symmetry between the left and right side muscles, with the center of pressure correctly distributed and avoiding any mandibular torque. Concerning the postoperative sequelae, all the patients reported hematoma and/or swelling but no major postoperative complications were observed.

## 4. Discussion

The EMG activity of the masticatory muscles in patients with maxillary deficiency and in patients with normal occlusion has been studied by different authors but with contradicting findings [26,27,35,36].

The correct functioning of the stomatognathic apparatus depends on the harmony created between all its components: jaw bones, temporomandibular joints, upper and lower dental arch, and masticatory muscles. Most of the studies present in the literature about palate expansion, whether surgically assisted or not, focus on the skeletal effects of treatments without considering the effect of these on the masticatory muscles [37].

Analysis of masticatory muscle activity in subjects with altered occlusal relationships, such as those who are candidates for SARME, could supply useful records on the functional impact of morphological discrepancies. These activities can be examined with surface electromyography (sEMG), which enables us to monitor some of the main masticatory muscles, with outcomes that do not differ significantly from those obtained with intramuscular recordings [38,39] and which have been found to have a good standard of reproducibility when carried out with appropriately standardized protocols [40,41].

SARME is an invasive surgical procedure that aims to solve transverse maxillary hypoplasia, greater than 5 mm, in adult patients. Once the treatment is completed, an adequate occlusal relationship with balanced muscle activity is expected, as well as an aesthetic and functional improvement [16,42,43].

Therefore, the goal of the present research was to analyze the changes in electromyographic activity of the masseter and temporalis muscles in skeletally mature patients before and after SARME to ascertain how this operation is able to influence the masticatory muscle activity after obtaining a new occlusal relation.

To the best of our knowledge, only one pilot study is present in the literature assessing the behavior of the masticatory muscles after SARME [34].

The electromyographic analysis performed by Sverzut et al. [34] showed that the activity of the masticatory muscles decreased in a significant way after SARME both at rest and during dynamic movements. The difference between the results obtained from our study could be explained by the fact that the second electromyographic analysis was carried out at two different times between the two studies: in the study by Sverzut et al. the follow-up was carried out 15 days later, whereas in the present study it was 6–9 months after surgery. After a longer interval, the results of SARME should be more stable as the musculature has more time to adapt to the new conditions of the stomatognathic system, both skeletal and functional. In fact, the patients reported similar electromyographic values at T1 and T0, maintaining the same muscular balance as before SARME.

The electromyographic indices used in this study (POC, CT, and Ac) are intended to highlight the importance that occlusion has on neuromuscular balance; a correlation already found by other authors in the literature [44,45].

Analyzing the values obtained in this study and shown in Table 2, it can be seen that all the electromyographic indexes analyzed are within the predetermined ranges at both T0 and T1.

This demonstrates how the neuromuscular system can be stable even in the presence of a crossbite and how 6–9 months after SARME the balance of the temporalis and masseter muscles is unaltered.

Comparable results were obtained from studies that analyzed the effect of RME on the EMG activity of the masticatory muscles in growing patients [35,46]. Both Arat et al. [47] and Di Palma et al. [35] found that 3 months after maxillary expansion with RME, the EMG potentials of the masticatory muscles returned to the values recorded before the treatment, demonstrating that the masticatory muscles can adapt well to maxillary expansion. In contrast, De Rossi et al. [26] showed that the EMG activity of the jaw muscles after RME increased compared with the pre-treatment results. The specific condition of the study may have influenced the findings as they used a bonded rapid maxillary expander with acrylic occlusal covering. Moreover, Michelotti et al. [27] evaluated an eventual asymmetric activation of the masticatory muscles in young subjects, concluding that the unilateral posterior crossbite does not take part in an asymmetric activation of the right and left superficial masseter and anterior temporalis muscles during functional tasks. This would explain the normal values highlighted at T0 in our sample.

The objective of the current research was not to compare normal patients with those presenting posterior crossbites but to prove the muscular alterations associated with SARME. Thus, no controls regarding normocclusive subjects have been included and each subject acted as a control of her/himself.

The limited sample size, but sufficient for a statistically significant comparison of the data, did not allow us to make a conclusive assertion on this topic, even if the use of standardized EMG potentials increases the significance of our findings.

Within the main limitations of this study, we can also include the lack of an analysis of dynamic movements as only the static neuromuscular coordination of patients was considered. Additional information could therefore be obtained by enlarging the number of patients, including an intermediate follow-up, and by also assessing jaw movements with motion analysis tools [48]. Furthermore, long-term EMG assessments were not considered. Therefore, future prospective studies including a longer follow-up and a larger sample size are necessary to obtain more reliable information on this subject.

## 5. Conclusions

Based on the specific limits of this study, it can be asserted that SARME did not modify the EMG activity of the masticatory muscles. The present study has shown that the temporal and masseter muscles were not influenced by this treatment and the new occlusal condition was related to symmetric muscular activities, at least during static conditions and in adults with satisfying muscular coordination before surgery.

## Figures and Tables

**Table 1:** For each patient, the following sEMG indices of neuromuscular coordination have been assessed.

Indices	
Percentage overlapping coefficient (POC, unit: %)	An index of the symmetric distribution of muscular activity as determined by occlusion. Calculations were performed for each pair of muscles, resulting in a POC for the temporalis muscles (POCt) and a POC for the masseters (POCm).
Torque coefficient (TC, unit: %)	Index of the possible presence of a mandibular torque.
Activity Index (Ac, unit: %)	Index suggesting the prevailing area of occlusal contacts.
Standardized activity (unit: μV/μV s %)	The total standardized electric activity developed by the muscles investigated during the maximum voluntary clamping (MVC) was calculated as the average integrated areas of temporalis and masseter Superficial electromyographic (EMG) potentials over time.

**Table 2:** Mean, standard deviation (S.D), and statistical comparison between pre- and post-treatment EMG values.

Variable		T0	T1	Difference	*p*-Value
POC t (%)	Mean	86.52	85.52	1	0.317
	S.D	3.75	5.73	3.38	
POC m (%)	Mean	84.23	85.78	−1.55	0.561
	S.D	8.57	5.71	7.17	
TC (%)	Mean	8.91	8.23	0.68	0.521
	S.D	3.60	2.15	3.29	
Ac (%)	Mean	2.35	4.81	−2.46	0.533
	S.D	12.28	10.62	14.22	
Standardized activity (μV/μV s %)	Mean	133.69	132.87	0.82	0.925
	S.D	43.89	50.4	36.03	

## Data Availability

Data are available on reasonable request due to restrictions, e.g., privacy or ethics.
